# Scientific Discovery and Women's Health

**DOI:** 10.3201/eid1011.AC1011

**Published:** 2004-11

**Authors:** Polyxeni Potter

**Affiliations:** * Centers for Disease Control and Prevention, Atlanta, Georgia, USA

**Keywords:** Art and science, emerging infectious diseases, women's health, cover text, AIDS, HIV, malaria

**Figure:**
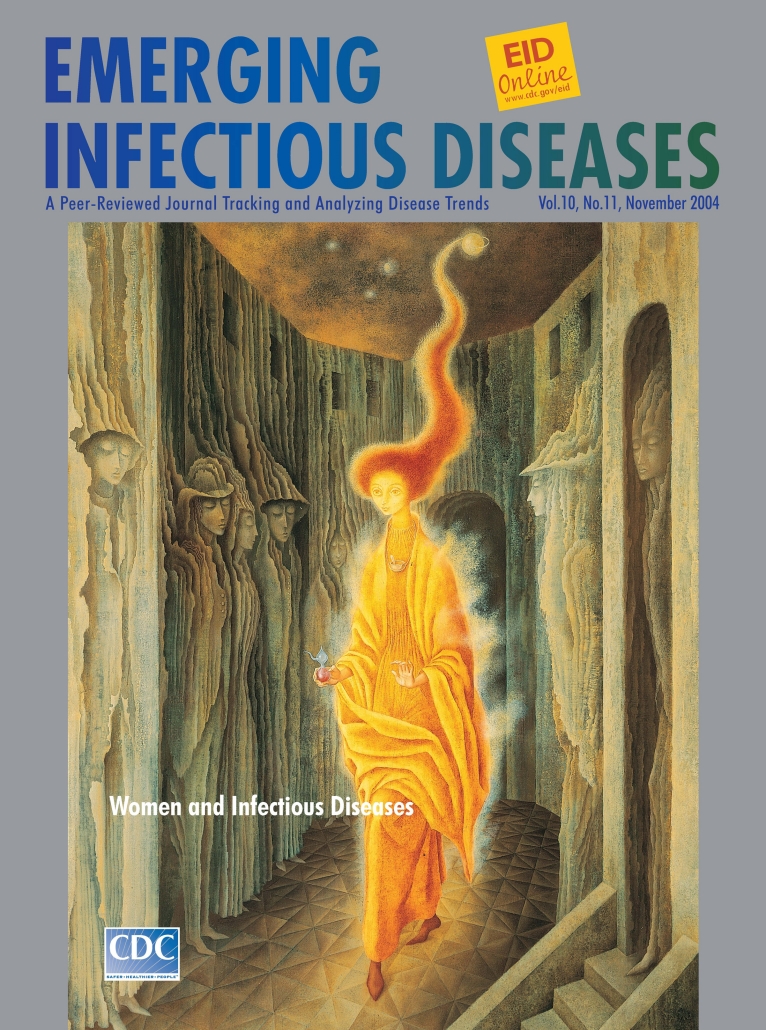
Remedios Varo (1908–1963). La Llamada (The Call) (1961) Oil on Masonite (98.5 cm x 68 cm). Private collection, courtesy of Walter Gruen

"Surrealism claims totally the work of the enchantress too soon gone," said André Breton, when he heard that Remedios Varo had died, in 1963 (*1*). Surrealism, which sought to express "the actual functioning of thought," was Varo's vehicle for understanding the universe, a vehicle that, like the fanciful locomotives in many of her paintings, went beyond established scientific principles. Bolstered by intuition and intellectual curiosity, the movement accessed the world of dreams, memory, and the psyche (*2*).

To this expansive world, Varo brought knowledge of engineering construction, painstaking attention to detail, a penchant for philosophical discourse, and fascination with alchemy and the occult (*3*). The result was a personal approach to surrealism, the unified vision of a fantastic world inhabited by creatures of the imagination, moving freely in and out of consciousness, proposing new solutions, offering alternative interpretations.

A native of Angles, Spain, Remedios Varo grew up in a family that nurtured academic and artistic aspirations. Her father, a hydraulics engineer, encouraged her interest in science and taught her how to draft images, a skill she used throughout her artistic career. At age 15, she enrolled in the renowned fine arts academy of San Fernando in Madrid, also attended around the same time by budding surrealist, Salvador Dalí.

At the academy, which featured such lecturers as Marie Curie, H.G. Wells, Albert Einstein, and José Ortega y Gasset, Varo became familiar with new ideas: the theories of Sigmund Freud, which broadened the boundaries of reality, the work of André Breton, which defined surrealism as a literary and artistic movement. She was exposed to the treasures of the Prado Museum and the influences of Hieronymus Bosch, Francisco Goya, El Greco, Picasso, and Braque.

Varo's rigorous academic training formed the backbone of an artistic career marked by innovation and creativity and frequently interrupted by conflict. The Spanish Civil War forced her to flee Barcelona, where she had become part of the bohemian avant-garde, for Paris, where she apprenticed among the surrealists' inner circle and exhibited her work widely. She left Europe to escape World War II, and Mexico became the adoptive home where in the last 10 years of her life she produced the bulk of her mature work.

Mexico, with its pre-Columbian cultures, primitive art, and abundant hospitality, provided Varo broad artistic freedom and an exciting context in which to practice surrealist rebellion. Yet, her first few years in exile were marked by economic hardship and emotional isolation: "We are finally installed here…suffering from the 2,400 meters altitude…dead with fatigue and having heart ailments" (*3*). Away from her familiar circle, she struggled to secure what Virginia Woolf once identified as the basic requirements for an artistic career: a steady income and "a room of one's own." She painted furniture, worked for Bayer Pharmaceuticals as illustrator, and during a brief visit to Venezuela, produced scientific drawings for that country's Ministry of Public Health (*4*).

Varo's interest in scientific discovery, reflected even in the titles of her works, extended to cosmology, evolution, astronomy, and genetics: The Phenomenon of Weightlessness, Cosmic Energy, Weaving of Space and Time, Creation of the Birds, Discovery of a Mutant Geologist, Exploration of the Sources of the Orinoco River, Vegetal Architecture. Her paintings showed empathetic understanding of the human condition and often contained elaborate mechanical devices and instruments of science meant to improve it.

"…as if she paints with her gaze rather than her hands, Remedios clears the canvas and over its transparent surface she gathers simple truths…" said Mexican poet Octavio Paz in his poem "Apparitions and Disappearances" (*3*). Her protagonists, who bear her heart-shaped face, almond eyes, long sharp nose, and abundant hair, move in a metaphysical world. As they straddle the line between real and unreal, they seem aware of their demands on the viewer's imagination. Witty and engaging, they levitate in narrative scenes filled with fantastic plant and animal life. Some cats are so wild they are made of ferns, some women so domesticated they have chair arms and chair legs.

The Call, on this month's cover of Emerging Infectious Diseases, is inhabited by apparitions and has the eerie stillness and depthless unreality of a dream. A flaming female figure charged by a celestial body emanates energy and lights up the scene; around her neck, a single ornament, a chemist's mortar; in her hand, a laboratory flask, a retort. The lurid presence casts a glow on the dim walls of a hallway. From these walls, like a hallucinogenic distortion, a mournful array of human forms bulge forward, feet anchored to the floor, eyes downcast, bodies lost in outlandish folds: female phantoms, pillars and structural support, trapped in a paralyzing nightmare.

Mysterious and provocative, the architectural stage is cluttered with conflicting clues. The walls are tall; the windows small and out of reach; the sky inflamed; the morbid folds props of oppression. Yet, the floor is elaborately tiled, the doorways arched, the steps well-tended. The stage is firmly cast, oppression is institutionalized.

Varo's enigmatic Call, part dream part symbolic reality, seems at once a calling and a call to action. The flaming figure wears the signs and halo of science. Bathed in the light of knowledge, she steps forward boldly to dispel the darkness. In the painter's surreal universe as well as ours, the female phantoms on the wall stand for poverty, confinement, disease. Overlooked by societies, biomedical research, and healthcare systems; battered by AIDS, malaria, and other infections; victimized by globalization; and stigmatized by the very diseases that confine and kill them (*5*), women slumber in the shadows. The flaming figure's flask contains the science. Her call is a wake-up call.
